# The Dynamics of B Cell Aging in Health and Disease

**DOI:** 10.3389/fimmu.2021.733566

**Published:** 2021-10-05

**Authors:** Jill de Mol, Johan Kuiper, Dimitrios Tsiantoulas, Amanda C. Foks

**Affiliations:** ^1^Division of BioTherapeutics, Leiden Academic Centre for Drug Research, Leiden University, Leiden, Netherlands; ^2^Department of Laboratory Medicine, Medical University of Vienna, Vienna, Austria

**Keywords:** B cells, aging, inflammaging, immunosenescence, autoimmune diseases, atherosclerosis

## Abstract

Aging is considered to be an important risk factor for several inflammatory diseases. B cells play a major role in chronic inflammatory diseases by antibody secretion, antigen presentation and T cell regulation. Different B cell subsets have been implicated in infections and multiple autoimmune diseases. Since aging decreases B cell numbers, affects B cell subsets and impairs antibody responses, the aged B cell is expected to have major impacts on the development and progression of these diseases. In this review, we summarize the role of B cells in health and disease settings, such as atherosclerotic disease. Furthermore, we provide an overview of age-related changes in B cell development and function with respect to their impact in chronic inflammatory diseases.

## Introduction

Aging has a major impact on the composition and function of the immune system, thereby drastically increasing the risk for inflammatory diseases (1). Not surprisingly, the ongoing demographic shift towards an older population results in an increased incidence of infections, autoimmune diseases and fatal cardiovascular events (2), underlining the importance to enhance our understanding of the age-associated changes in the immune system, which are termed immunosenescence.

During aging, hematopoietic stem cells (HSCs) in the bone marrow show reduced self-renewal and differentiate preferentially towards the myeloid cell subsets (3). As a result, the production of neutrophils and monocytes is increased, whereas the generation of B and T lymphocytes is drastically declined. In addition to alterations in the number of immune cells, aging hallmarks, including genomic instability, telomere shortening, epigenetic dysregulation and cellular senescence, contribute to the malfunctioning of the innate and adaptive immune system (4). Several studies showed that the innate immune response in aged mice is prolonged due to the decreased phagocytic ability by neutrophils and macrophages (5, 6). Moreover, aged dendritic cells (DCs) showed an increased secretion of the pro-inflammatory cytokines IL-6 and TNF-α (7). Aging is also associated with crucial changes in the adaptive immune response. Aged T cells displayed reduced proliferative capacity and an increased production of pro-inflammatory cytokines (8, 9), thereby contributing to a chronic state of low-grade inflammation, called ‘inflammaging’. The age-related defects in CD4^+^ T cell helper function also impaired B cell responses (10). Moreover, aging is associated with intrinsic B cell defects, such as reduced antibody production and decreased affinity maturation in antibody responses resulting in an increased risk for infections (11). In light of the current pandemic, this also contributes to the high infection rate and poor prognosis of COVID-19 in the aged population (12). Dysfunctional B cell responses in the elderly, including increased autoantibody production, are also associated with an increased risk for autoimmune diseases and other chronic inflammatory diseases, such as atherosclerosis (13). Taken together, B cells play a major role in these diseases *via* antibody secretion, antigen presentation and T cell regulation. This review aims to provide an overview of the effects of aging on the functions of B cells in health and disease settings.

## B Cell Development

B cells are antigen-presenting cells (APCs) that are generated from multipotent HSCs (14–16). In the bone marrow, HSCs differentiate into B lymphocyte progenitors, which further differentiate into progenitor B cells (pro-B cells), precursor B cells (pre-B cells) and immature B cells (17). These developmental stages can be distinguished by the expression of different markers on the cell surface (**Table 1**). During this process, each B cell clone develops a unique B cell receptor (BCR) with a specific epitope-binding site *via* sequential immunoglobulin gene recombination of variable, diversity and joining genes (32). The generated heavy and light chain polypeptides, which consist of constant and variable regions, form the mature BCR (33). Together with B cell-specific membrane proteins, including CD19, BCRs form signaling complexes that activate the NF-κB, PI3K and MAPK pathways (34). These pathways, in turn, stimulate cell survival and induce the migration of transitional immature B cells to the spleen for their final stages of maturation (35). Subsequently, mature B cells migrate to the peritoneal cavity or lymphoid follicles of secondary lymphoid organs, where they can encounter foreign antigens. Upon binding of an antigen to the BCR, in combination with innate and costimulatory signals, B cells can function as APCs and differentiate into antibody-secreting plasma cells (36, 37). Under infectious conditions, antigen-specific B cells present peptides *via* major histocompatibility complex (MHC) II to naive CD4^+^ T cells, resulting in CD4^+^ T cell activation and follicular helper T cell (T_FH_) differentiation (38). Moreover, multiple studies investigating the APC function of B cells showed that B cell-derived cytokines contribute to T cell profile skewing, such as T helper (T_H_) 1 or T_H_2 (39, 40). Most B cells, however, are activated *via* T cell-independent (TI) or T cell-dependent (TD) mechanisms (41). During T cell-independent (TI) B cell activation, antigens with repetitive epitopes, such as polysaccharides, bind to the BCR, resulting in BCR crosslinking (42). Together with costimulation from toll-like receptors (TLRs), this leads to the development of short-lived plasma cells. In contrast, T cell-dependent B cell activation results in long-lived plasma cell differentiation and memory B cell formation (43). This type of activation requires antigen presentation to and costimulation from T_H_2 or T_FH_ cells. In turn, B cell-derived plasma cells secrete immunoglobulins with heavy and light chains similar to the BCR in order to mark or neutralize the foreign antigen (44). Depending on the type of activated B cell, these antibodies can be of the IgA, IgD, IgE, IgG or IgM isotype (45).

**Table 1:** Phenotype of distinct B cell subsets in mice and humans.

Species	Organ	B cell subset	Markers	Literature
Mouse	BM	B lymphocyte progenitor	Ly6D^+^ IL-7Rα^+^ CD135^+^ CD3^-^ CD4^-^ CD8^-^ Gr-1^-^ CD11b^-^ TER-119^-^ CD117^-^ Sca-1^-^	(15, 17)
		B-1 progenitor	CD19^+^ CD93^+^ CD3^-^ CD4^-^ CD8^-^ Gr-1^-^ CD11b^-^ TER-119^-^ B220^low^	
		B-2 progenitor	B220^+^ CD3^-^ CD4^-^ CD8^-^ Gr-1^-^ CD11b^-^ TER-119^-^ CD19^-^ CD93^-^	
		Pre-Pro B cell	B220^+^ CD43^+^ CD93^+^ CXCR4^+^ CD135^+^ IL-7Rα^+^ CD3^-^ CD4^-^ CD8^-^ Gr-1^-^ CD11b^-^ TER-119^-^ CD19^-^ CD24^low^ CD117^-^ IgM^-^	
		Pro-B cell	B220^+^ CD19^+^ CD24^+^ CD43^+^ IL-7Rα^+^ CD3^-^ CD4^-^ CD8^-^ Gr-1^-^ CD11b^-^ TER-119^-^ CD117^-^ IgM^-^	
		Pro-B cell	B220^+^ CD19^+^ CD24^+^ IL-7Rα^+^ CD3^-^ CD4^-^ CD8^-^ Gr-1^-^ CD11b^-^ TER-119^-^ CD43^-^ IgM^-^	
		Immature B cell	B220^+^ CD19^+^ CD24^+^ CD93^+^ IgM^+^ CD23^-^ CD43^-^ IgD^-^	
	Peritoneal Cavity	B-1a cell	CD19^high^ CD43^+^ CD1d^mid^ CD5^+^ CD23^-^	(18, 19)
		B-1b cell	CD19^high^ CD43^+^ CD1d^mid^ CD23^-^ CD5^-^	
	Spleen	T1 B cell	B220^+^ CD19^+^ CD24^+^ CD93^+^ IgM^+^ CD43^-^ IgD^low^	(15, 17)
		T2 B cell	B220^+^ CD19^+^ CD24^+^ CD93^+^ IgM^+^ IgD^+^ CD43^-^	
		MZ B cell	B220^+^ CD19^mid^ CD21^high^ CD1d^+^ IgM^high^ CD43^-^ CD23^-^ CD93^-^ IgD^low^	(20, 21)
		FO B cell	B220^+^ CD19^mid^ CD23^high^ CD1d^mid^ IgD^high^ CXCR5^+^ CD43^-^ CD21^low^ IgM^low^	
		ABC	CD19^+^ BAFFR^+^ CD11b^+^ CD11c^+^ (T-bet^+^)	(22, 23)
	Lymphoid Tissue + Peripheral Blood	GC B cell	B220^+^ CD19^+^ CD40^+^ MHCII^+^	(24)
		B_REG_	CD19^+^ CD1d^high^ CD5^+^	(25)
Human	BM	B lymphocyte progenitor	CD10^+^ CD34^+^ Pax5^+^	(16)
		Pre-Pro B cell	CD10^+^ CD34^+^ Pax5^+^ CD38^+^ CD117^-^	
		Pro-B cell	CD10^+^ CD34^+^ Pax5^+^ CD38^+^ CD19^+^ CD20^+^ CD24^+^ CD93^+^ IL-3R^+^ IL-7Rα^+^ CD117^-^	
		Pre-B cell	CD10^+^ Pax5^+^ CD38^+^ CD19^+^ CD20^+^ CD24^+^ CD93^+^ IL-3R^+^ IL-7Rα^+^ IL-4Rα^+^ CD117^-^ CD34^-^	
		Immature B cell	CD10^+^ CD38^+^ CD19^+^ CD20^+^ CD24^+^ CD93^+^ CD21^+^ CD40^+^ IL-4Rα^+^ CD117^-^ CD27^-^ IL-7Rα^-^	
	Peritoneal Cavity	B-1 cell	CD20^+^ CD27^+^ CD43^+^ CD38^low^	(26)
	Spleen	Transitional B cell	CD38^+^ CD19^+^ CD20^+^ CD24^+^ CD93^+^ CD21^+^ CD23^+^ CD5^+^ TACI^+^ CD10^-^ CD27^-^	(16)
		MZ B cell	CD19^+^ CD20^+^ CD21^+^ TACI^+^ CD1c^+^ CD27^+^ FCRL3^+^	(27)
		FO B cell	CD19^+^ CD20^+^ CD21^+^ TACI^+^ CD22^+^ CD23^+^ CXCR5^+^ MHCII^+^ CD10^-^ CD27^-^ CD38^low^ CD24^low^	
		ABC	CD19^+^ BAFFR^+^ CD11b^+^ CD11c^+^ (T-bet^+^)	(28, 29)
	Lymphoid Tissue + Peripheral Blood	GC B cell	CD38^+^ CD19^+^ CD20^+^ TACI^+^ CD27^+^ MHCII^+^ CD40^+^ CD83^+^	(30)
		B_REG_	CD19^+^ CD24^high^ CD27^+^	(31)

### B-1 Development

In the early stages of B cell development, two distinct lineages arise from different precursor cells (46). The B-1 lineage, which was originally studied in mice where it develops into the B-1a and B-1b subsets, is predominant in neonates and is characterized by the expression of CD43 (18, 26). Although the exact mechanisms of B-1 development are not fully understood, the absence of nucleotides at the junctions of V and J segments in their BCR (47), which are inserted in other B cell subsets by the postnatal enzyme terminal deoxynucleotidyl transferase (48), suggests that B-1 cells are originally derived from fetal liver progenitor cells. Some studies indicated that B-1 progenitors develop prior to the existence of HSCs from pre-HSCs or fetal multipotent progenitor cells (49, 50). However, it has also been shown that fetal HSCs are responsible for B-1 development and that fetal lymphopoiesis can be restored in adult HSCs with Lin28b overexpression (51), suggesting that B-1 cells are generated in different waves. B-1 cells mainly reside in the peritoneal cavity where they can elicit antibody-producing and antigen-presenting functions (52). B-1a cells, that in contrast to B-1b cells also express the T cell surface glycoprotein CD5 (19), are responsible for most naturally occurring IgA and IgM antibodies, which are produced in the absence of antigen stimulation (53). Upon infection, B-1a cells can also respond to TI antigens and produce pathogen-specific antibodies (54). B-1b cells, however, have a broader antigen repertoire which allows them to produce bacteria-specific IgM in response to TI antigens and generate TI memory (55). In addition to antibody production, B-1 cells can activate CD4^+^ T cells through antigen presentation (56). Contradictive results are shown regarding the predominance of B cells in CD4^+^ T cell activation, and thus the exact role of B cells in antigen presentation is not fully understood (38, 57, 58). It has been previously reported that B-1a cells present antigens to both peritoneal and peripheral CD4^+^ T cells (59). In the peritoneal cavity, B-1a cells stimulate the activation of IL-10-, IL-4- and IFN-γ- producing T cells, whereas splenic B-1a cells induce T_H_17 differentiation (60). Splenic and serosal B-1a cells can also transform into granulocyte macrophage-colony stimulating factor- (GM-CSF) producing innate response activator (IRA) B cells (61). In addition to GM-CSF, which stimulates extramedullary hematopoiesis and DC activation during chronic inflammation (62), IRA B cells secrete IgM and IL-3, which in turn promotes monocyte and neutrophil production (63).

### B-2 Development

B-2 cells comprise most of the adult B cell population in peripheral tissues. In contrast to the B-1 subset, mature B-2 development and survival is dependent on the B cell activating factor (BAFF) – BAFF receptor (BAFFR) signaling pathway (64). Expression of the pro-survival receptor BAFFR starts when transitional B-2 cells undergo their final maturation stages in the spleen (65). BAFF is expressed by various cell subsets as monocytes, macrophages, DCs, stromal cells and T cells (66). Disruption of BAFF-BAFFR signaling results in dramatically decreased B-2 cell numbers (67).

B-2 cells can be further subdivided in marginal zone (MZ) and follicular (FO) B cells (68). MZ B cells are formed upon Notch-BRP-J signaling and are distinguished by very low CD23 and high CD21 expression (20, 21, 27). Upon recognition of a pathogen, short-lived MZ-derived plasma cells secrete high volumes of IgM. In addition, MZ B cells have the capacity to phagocytose the invading pathogen and present antigens to naive CD4^+^ T cells (69). Antigen presentation by MZ B cells induces T_H_1 effector differentiation and might thus be important for the generation and reactivation of memory CD4^+^ T cells (70). After TD activation, FO B cells can ultimately differentiate into plasma cells, which are long-lived and produce high amounts of antibodies (71). However, the majority of FO B cells interact with antigen-specific T_FH_ cells to become GC B cells (24), which express high levels of the DNA-mutating enzyme activation-induced cytidine deaminase (AID) (30). Under the influence of CD40 ligand-mediated signals and cytokines, secreted by T_FH_ cells, AID becomes activated and induces class switch recombination (CSR) (72). During CSR, the constant region of the BCR is hypermutated and as a result, the antibody production of the GC B cell is switched from IgM to IgG, IgA or IgE isotypes (73).

### Regulatory B Cells

There is evidence that some B cell subpopulations have the capacity to differentiate into regulatory B cells (B_REGS_) under pro- and anti-inflammatory environmental conditions (74). Although the exact source of B_REGS_ remains elusive, the similarity of cell surface markers between B_REGS_ and various B cell subsets indicates a role for BCR signaling rather than the existence of a specific B_REG_ precursor (75). The development of B_REGS_ is antigen-specific and can be induced *via* innate and adaptive mechanisms (76). In the adaptive pathway, B_REGS_ present antigens to CD4^+^ T cells and become activated through CD40-CD40L and IL-21 signals (77), whereas in the innate pathway, TLR2 and TLR9 signaling and IL-1β play important roles (78). In addition, B_REGS_ can be induced by the anti-inflammatory cytokine IL-35 (79). B_REGS_ can have different phenotypes (**Table 2**) and provide tolerance *via* various IL-10-dependent and IL-10-independent mechanisms (85) including cell-cell contact and the secretion of IL-35 and TGF-β (86–88). The IL-10-producing B10 subset expresses CD1d in mice and CD24 in humans (25, 31). In both species, B10 cells suppress the APC function of DCs and inhibit T_H_1 and T_H_17 differentiation. A B_REG_ subset that inhibits effector T cells and promotes T_REG_ differentiation through PD-L1 interaction was also identified in both mice and humans (81). Murine TIM-1^+^ B_REGS_ also secrete IL-10 and mainly promote T_H_2 and T_REG_ generation (80). Moreover, Kaku et al. showed that some murine B_REGS_ mediate their immunosuppression *via* CD73 expression and adenosine production (82). Interestingly, in humans, a CD73^low^ B cell subset was marked as CD4^+^ T cell-suppressing B_REG_ (84). Transitional B cells, B-1a and MZ B cells can also suppress CD4^+^ and CD8^+^ T cell activation and are therefore sometimes classified as B_REGS_. In addition, Lundy et al. discovered that some B-1a cells exert their tolerogenic effects *via* the expression of the apoptotic surface molecule FasL (83). Further studies are required to elucidate if distinct B_REG_ subpopulations are indeed derived from specialized conventional B cell subsets or whether B_REGS_ derive from specific progenitor cells and differentiate into subsets after encounter with an antigen.

**Table 2:** Phenotypes and functions of regulatory B cells.

Species	B_REG_ subset	Markers	Function	Literature
Mouse	B10 cells	CD19^+^ CD5^+^ CD1d^+^	Induce T_REGS_ and inhibit T_H_1 and T_H_17 differentiation	(25)
	TIM-1^+^ B cells	CD19^+^ TIM-1^+^	Promote T_H_2 and T_REG_ differentiation	(80)
	PD-L1^+^ B cells	CD19^+^ PD-L1^+^	Promote T_REG_ differentiation	(81)
	CD73^+^ B cells	B220^+^ CD39^+^ CD73^+^	Inhibit effector T cells	(82)
	B-1a FasL^+^ cells	CD19^+^ CD5^+^ FasL^+^	Mediate CD4^+^ T cell apoptosis	(83)
Human	B10 cells	CD19^+^ CD24^high^ CD27^+^	Induce T_REGS_ and suppress T_H_1 and T_H_17 differentiation	(31)
	PD-L1^+^ B cells	CD19^+^ PD-L1^+^	Suppress pro-inflammatory cytokine production and inhibit CD8^+^ T cell activation	(81)
	Br1 cells	CD25^high^ CD71^high^ CD73^low^	Inhibit CD4^+^ T cell proliferation	(84)

## Aging and B Cell Subsets

Recent studies revealed that the production of precursor B cells in the bone marrow is significantly decreased in aged mice and humans (89–92). This reduction could be attributed to age-related changes in the microenvironment of the bone marrow, including diminished levels of the pro-B cell-survival cytokine IL-7 and an enlarged bias of HSCs to produce myeloid cells instead of lymphocytes (93, 94). Despite the comparable reduction in precursor B cells in both mice and humans, conflicting results regarding peripheral B cells have been shown. In mice, the number of mature splenic B cells is maintained due to an increase in autoreactive and age-associated B cells (ABCs) (95). Although the ABC subset is also detected in humans (96), the absolute number of B cells in the periphery declines with age in humans (97). In the study of Frasca et al., the number of peripheral naive B cells was unaffected, whereas the absolute number of switch memory B cells declined upon aging. Noteworthy, the characterization of memory B cells in mice and humans is not identical. According to Reynaud et al., recirculating MZ B cells in humans are defined as long-lived IgM memory B cells in mice (98). Additionally, Benitez et al. illustrated that the dynamics of peripheral B cell production differs between humans and mice (99). In particular, the splenic FO B cell compartment was larger in humans compared to mice, which might explain the difference in observed effects of aging on B cell numbers. Nevertheless, the quality of aged peripheral B cells is reduced in both mice and humans. Frasca et al. discovered that aging downregulates CSR in human and murine peripheral B cells (97, 100), which might explain why aged murine plasma cells mainly secrete IgM, whereas young murine plasma cells mainly secrete IgG (101). Antibodies generated in aged mice also showed decreased affinity and thus provided less protection (102). Although these studies mainly investigated IgG responses, aging affects all B cell subsets (**Table 3**).

**Table 3:** Effects of aging on B cell subsets.

B cell subset	Model	Effect of aging	Literature
Total B cells	Young (2-3 months) and old (>8 months) C57BL/6 and BALB/c mice	Reduced numbers of B cell precursors	(89, 91)
	Human bone marrow specimens from 598 patients (2 months – 98 years)	Decreased numbers of B cell precursors and changed antibody expression	(92)
	Human peripheral blood from 46 donors (18-86 years) and young (2-4 months) and old (24-27 months) BALB/c mice	Impaired CSR	(96, 97)
	Human peripheral blood from 130 donors (21-99 years)	Declined percentage of memory B cells, no effect on total number of peripheral B cells	(97)
	Young (2 months) and old (24 months) C57BL/6 mice	Increased IgM production, decreased IgG production	(101, 102)
B-1 cells	Young (2-4 months) and old (21-26 months) BALB/c mice	No effect on B-1 precursors	(103)
	C57BL/6 and C57BL/6 SCID mice (1-18 months)	Increased number of peritoneal B-1b cells	(104)
	Human peripheral blood from 85 donors (20-103 years)	Decreased percentage of CD5^+^ B cells	(105)
	Studies of vaccination against pneumococcal infections in elderly (65+)	Reduced protection of antibodies secreted by B-1 cells against bacteria	(106)
	PBMCs from young (~ 41 years old) and old (~79 years old) individuals and young (5-8 weeks) and old (18 – 22 months) C57BL/6 mice	Accumulation of 4-1BBL^+^ MHC-I^+^ CD86^HI^ B cells	(107, 108)
	Young (2-3 months) and old (16-22 months) BALB/c and C57BL/6 mice	Reduced B-2 antibody production to T-dependent antigens	(109)
B-2 cells	Young (2-3 months) and old (20-25 months) C57BL/6 mice	Dysfunctional antibody production	(110, 111)
	Young (2-4 months) and old (21-26 months) BALB/c mice	Reduced number of B-2 progenitors	(112)
	Young (3 months) and old (18 months) C57BL/6 mice	Reduced number of FO B cells, increased sensitivity of MZ B cells to BAFF	(113)
	Young (2-4 months) and old (>20 months) B10.BR mice	Impaired GC expansion and differentiation and reduced CD4^+^ T cell helper function	(114)
	Young (2-3 months) and old (20-21 months) BALB/c mice	Impaired FO DC function, reduced GC formation and B-2 antibody production	(115)
	Human peripheral blood from 85 donors (20-103 years)	Decreased numbers of naive and memory B-2 cells, increased percentage of unswitched IgM memory cells	(105)
	Human peripheral blood from 54 donors (20-45 and 70-86 years)	Reduced percentage of classical switched memory B cells	(116)
	Young (3 months) and old (10 months) SAMP8 mice and young (2 months) and old (17-18 months) BALB/c mice	Reduced number of MZ B cells and inhibited T-independent antibody responses	(117, 118)
	C57BL/6 mice (2-30 months)	Impaired antigen capture and immunoglobulin production of MZ B cells	(119)
ABCs	Young (2-3 months) and old (>22 months) B10.D2 mice	Increased amount of peripheral antigen-experienced B cells	(95)
	Young (2-5 months) and old (24-29 months) C57BL/6 mice	ABC expansion and decreased number of IL-10 secreting FO B cells	(120)
	C57BL/6 and C57BL/6 × BALB/c mice (3-22 months)	Increased number of ABCs, probably due to exhausted FO expansion	(121)
	Young (3-4 months) and old (>20 months) C57BL/6 mice	Depletion of ABCs in aged mice revived B cell production	(122)
	Young (1-3 months) and old (>24 months) C57BL/6 mice	Accumulation of ABCs in female mice	(28)
	Human peripheral blood from 88 donors (20-55 and 75-102 years)	Elevated percentage of double negative exhausted memory cells	(123)
	Young (2 months) and old (15 months) C57BL/6 mice	Increased proportion of T_H_17 cells*	(124)
AABs	Young (3-4 months) and old (18-24 months) C57BL/6 mice	Accumulation of AABs in the VAT of obese mice	(125)
	Young (3 months) and old (19-24 months) C57BL/6 mice	AAB accumulation in the VAT of obese female mice	(126)

*Possible indirect effect of ABCs.

### Aging and B-1 Cells

As mentioned above, most B-1 cells are derived from fetal or neonatal precursors and are maintained *via* self-replenishment during the adult life (127). According to Alter-Wolf et al., the number of B-1 progenitors in mice is unaffected upon aging and Hinkley et al. even observed an increase in murine B-1 cells due to clonal expansion (103, 104). In contrast, studies in young and aged humans showed that the percentage of B-1 cells is decreased in the elderly (105, 128), which could possibly be explained by the low capacity of the adult human bone marrow to generate B-1 cells compared to adult murine bone marrow. While the antibody-secreting capacity remained unaffected in humans, the number of spontaneously IgM-secreting B-1 cells was reduced most likely *via* downregulation of the transcription factors XBP-1 and Blimp-1, both involved in the proliferation and differentiation of plasma cells (129, 130), and upregulation of the plasma cell-inhibiting transcription factor PAX-5 (131). In addition to the decrease in IgM-secreting B-1 cells, the diversity of IgM antibodies was reduced (103). In line with these findings, Adler et al. illustrated that aging limited bacterial protection by IgM secreted by B-1 cells (106). This could be the effect of N-region nucleotide additions, which are found on immunoglobulins produced by aged B-1 cells (132). Together these results suggest that B-1 cell functions in humans are impaired upon aging. Although the number of B-1 cells was not decreased in aged mice, recent research showed that aging affected B-1a cells (107). Under the influence of aged myeloid cells, B-1a cells in aged mice and humans were converted into pro-inflammatory 4-1BBL expressing cells. These 4-1BBL^+^ B cells were shown to activate anti-tumor granzyme B-secreting CD8^+^ T cells *via* the MHC/TCR and 4-1BBL/4-1BB axes (108). This finding implies that aging also affects murine B-1 cell functionality. However, Lee-Chang et al. only studied the effects of aging on neonatal B-1a cells. Since generation of the B-1b subset seems to be more predominant in aged mice (104), B-1b cells should be further investigated in an aged setting.

### Aging and B-2 Cells

Since Hu et al. reported in 1993 that aging reduced the B-2 cell antibody response to T cell-dependent antigens in mice, the effects of aging on the B-2 subset are under broad investigation (109). Initially, the loss of IgG and high affinity antibodies was thought to be induced by dysfunctional aged DCs and T cells (10, 133). DCs and T cells of aged mice were shown to be defective in the expression of B cell-activation markers, such as CD40L, resulting in diminished numbers of GC B cells and impaired isotype switching. Moreover, the number of regulatory T cells (T_REGS_), which can directly suppress B cell functions, is increased in aged mice (134, 135). Although contradicting results regarding the age-related effects on the immunosuppressive activity of T_REGS_ has been shown, Sage et al. reported that the frequency and function of follicular T_REGS_ was increased in aged mice (136), resulting in a defective antibody response. However, an adoptive transfer with young T cells and aged B cells to immunodeficient mice also resulted in low levels of high affinity antibodies, implying intrinsic B cell defects (110). Consistent with this finding, Frasca et al. concluded that isotype switching was impaired in aged B-2 cells in both mice and humans due to a reduction in E47 mRNA stability and AID transcription (111). In addition to the reduced antibody affinity, the number of progenitor cells is decreased in aged murine bone marrow (112). Despite the maintenance of peripheral B-2 cell numbers in mice, the amount and percentage of the FO B cell subset is slightly declined upon aging (113). Together with the disrupted follicle organization of aged T cells (114), this might explain the reduction in GC numbers (115). The reduction in the FO B cell subset might also be responsible for the decline in naive and memory B-2 cell numbers in aged humans (105, 116). Apart from the FO lineage, aging also affects the MZ B cell subset. Although the exact effects of aging on MZ B cells remain unclear, recent studies reported an age-associated increase in autoreactive antibodies (137). Since previous research illustrated that autoreactive MZ B cells might elude negative selection in the presence of high levels of the pro-survival factor BAFF (138), which is increased upon aging (139), Miller et al. speculated that the increase in autoreactive antibodies is caused by BAFF hypersensitivity of aged MZ B cells (113). However, Miller et al. did not investigate MZ B cell numbers in aged mice. In contrast to the hypothesis by Miller et al., both Cortegano and Birjandi et al. observed a reduction of MZ B cells in aged mice (117, 118). The age-related disruption of the marginal zone due to a decline in MZ macrophages may partially account for the reduction in MZ B cells. Besides decreased MZ B cell numbers, Turner et al. showed that antigen capture and antibody production of aged MZ B cells was defective, resulting in a diminished T cell-independent immune response (119). Altogether, these studies suggest that aging impairs B-2 cell functions in both humans and mice.

### Age-Associated B Cells

The maintenance of B-2 cell numbers in aged mice could possibly be explained by the accumulation of age-associated B cells. In 24 to 30 months-old C57BL/6 mice, half of all splenic B cells are considered to be ABCs, whereas the frequency of this population is extremely low in young mice (120). These ABCs are observed in both mice and humans and could be distinguished from other B cell subsets by their expression markers, including CD11b, CD11c and T-bet, and innate activation stimuli, such as toll-like receptor TLR7 signals (22). Although the exact cell surface markers are still under debate, ABCs do not express CD43 and CD5 that are found on B-1 cells. The ABC subset is also different from MZ and FO B cells, since ABCs do not depend on BAFF signals (121). HoweverHao et al. did show that FO B cells can transform into ABCs in both young and aged mice, suggesting that ABCs are derived from B-2 cells (121). This might also explain the reduction in the FO B cell pool upon aging (113). When Knode et al. repeated this experiment in aged mice, they concluded that ABCs arise from GC B cells following T cell-dependent antigen activation (140), thereby indicating ABCs as memory subset. In contrast, recent studies have shown that ABCs mainly arise in an antigen-limited environment and proposed that homeostatic expansion stimulates ABC formation to balance B cell progenitor loss (141–143). However, ABCs were reported to inhibit B cell generation *via* the production of TNF, and their depletion resulted in reactivated B lymphopoiesis (122, 144), suggesting that the loss of B cell precursors is caused rather than balanced by ABCs. Nonetheless, different subsets of ABCs have been described, indicating that ABCs can be generated *via* different developmental routes dependent on environmental factors, such as age and antigen load. One of these subsets, comprising almost two-thirds of all ABCs, is characterized by the expression of the T_H_1 transcription factor T-bet (23, 28). In addition, a T-bet negative subset, which is characterized by the expression of CXCR5, has been reported (29). The expansion of the T-bet^+^ subset is dependent on innate stimuli, such as TLR7 and TLR9 ligands, in an IL-21 and IFN-γ rich environment. Previous research demonstrated that the transient expression of T-bet by B cells stimulated IgG2a antibody isotype switching and a correlation between ABCs and protective IgG2a was also observed in virally infected mice (145, 146). In humans, an increase in ABCs was associated with elevated levels of IgG1 (147). Notably, Colonna-Romano et al. discovered the expansion of an ABC-like CD27^-^ IgD^-^ IgG^+^ memory B cell subset in the periphery of aged individuals, clarifying the high numbers of IgG observed in the elderly (123). Although T-bet expression was not shown for the latter double negative memory subset, Wang et al. proposed that loss of T-bet is probably necessary for the conversion of ABCs into plasma cells (148), suggesting that the double negative memory B cell might be an intermediate state from ABC to plasma cell. However, if and how plasma cells can be derived from ABCs remains poorly understood. Nonetheless, ABCs are speculated to secrete autoreactive antibodies, explaining their abundance in autoimmune diseases (149). Aside from immunoglobulin production, the antigen presenting function of ABCs is intensively investigated. Whereas Colonna-Romano et al. reported that the double negative exhausted memory B cell is incapable of antigen presentation, recent findings conclude that ABCs are efficient APCs that skew T_H_17 differentiation, thereby possibly explaining the increase in T_H_17 differentiation observed in aged mice and humans (124, 150, 151). Upon activation, T_H_17 cells secrete the pro-inflammatory cytokine IL-17, which plays important roles in both protective immunity and autoimmunity by the induction of neutrophil migration and activation (152), thereby contributing to inflammaging. Moreover, ABCs promote inflammaging by the production of IFN-γ and TNF-α (144). Strikingly, ABCs were also observed to secrete high levels of the anti-inflammatory cytokine IL-10, suggesting an immunoregulatory function. Ratliff et al., however, stated that this IL-10 was produced by an ABC-inhibiting regulatory FO B cell subset (120). Although many studies confirm the presence of an ABC subset in both mice and humans, the expression markers defined in these studies differ considerably. Some studies include CD11c and T-bet, whereas others focus on CD21 and CD27 or the production of IgM and IgG. These differences indicate heterogeneity in the ABC subset, suggesting that further research should examine age-related effects on distinct ABC subsets.

### Adipose-Resident Aged B Cells

In the white visceral adipose tissue (VAT) of aged female obese mice, Frasca et al. discovered the accumulation of an exhausted memory B cell subset similar to ABCs (125). In addition, Frasca et al. highlighted that FO B cells in a fatty environment rapidly transform into this pro-adipogenic B cell subset, resulting in a disrupted lipid metabolism. Comparable to splenic ABCs, this subset, defined as adipose-resident age-related B cells (AABs), expressed pro-inflammatory markers and was found to be more abundant in aged female than in aged male mice (126). Since the sex-related differences in ABC content are thought to be caused by TLR7, which is located on the X-chromosome and has been shown to escape X-chromosome silencing (153), the activation mechanisms of AABs are thought to be similar to ABCs. Although the direct effects of TLR7 signaling on AAB accumulation are not investigated, Camell et al. concluded that AAB expansion is dependent on the NLRP3 inflammasome (126), which can be activated by TLR7 signaling (154). Interestingly, upon NLRP3 deficiency, Camell et al. observed a reduction in both VAT AABs as splenic ABCs, confirming comparable activation routes for these subsets. In contrast, AABs do not express T-bet and induce T_H_1 differentiation rather than T_H_17 polarization (155, 156), indicating that AABs are functionally different from ABCs. Altogether, these data illustrate that aging induces the expansion of ABCs in the spleen and the accumulation of AABs in white VAT, resulting in impaired antibody responses and disrupted lipolysis.

### Aging and B_REGS_

As observed by Ratliff et al., aging reduced the number of an IL-10-secreting regulatory FO B cell subset (120) and similar to these findings a reduction in IL-10 levels upon aging was reported recently (157). However, IL-10 is not exclusively produced by B_REGS_ and the effects of aging on B_REGS_ are thus far not examined. The general decrease in B cell precursors and B-1 and B-2 subsets implies a subsequent reduction in B_REGS_. Nevertheless, the immunosuppressive functions of T_REGS_ were observed to be increased (158), whereas naive T cell numbers decreased upon aging (159). Since the differentiation of naive T cells into T_REGS_ can be stimulated by B_REGS_ (160), the immunosuppressive function of B_REGS_ is possibly also augmented upon aging. Contributing to this hypothesis, Dang et al. showed that PD-L1 expression on B lymphocytes increased with aging in healthy donors (161). However, these PD-L1-expressing B cells were identified by CD19 only and not specifically defined as B_REGS_. In contrast, increased autoimmunity in the elderly might suggest an age-related loss of tolerogenic B_REGS_ (162). Since B_REGS_ play an important role in regulating inflammaging and balancing the pro-inflammatory responses of ABCs, it is necessary to investigate the consequences of aging on the number and functions of the B_REG_ subset.

## Aged B Cell Homeostasis in Disease

### The Aged B Cell in Infections

**Figure 1:**
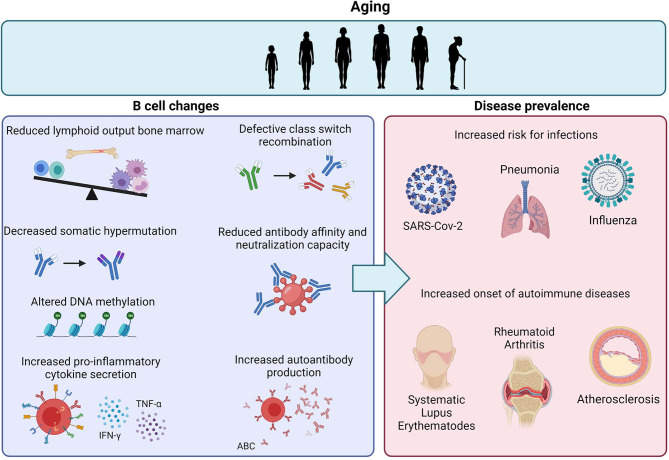
Age-related effects on B cells and the impact on disease prevalence. Aging is associated with reduced lymphoid output in the bone marrow and intrinsic defects in B cells, including decreased CSR, SHM and antibody affinity. The increase in ABCs also results in increased pro-inflammatory cytokine secretion and autoantibody reduction. Together, these age-related B cell changes contribute to the increased risk for infections and autoimmune diseases upon aging.

The age-associated changes in the immune system heavily increase the risk for bacterial and viral infections in the elderly (**Figure 1**) (163). In combination with the reduced responses to vaccinations in the elderly, this increase results in high hospitalization and mortality rates due to infectious diseases, such as COVID-19, pneumonia and influenza (164, 165). Although aging of the T cell repertoire and the associated T_H_1/T_H_2 imbalance, with a reduced T_FH_ and T_H_2 output in the elderly (136, 166), contributes to the high frequency of influenza infection (167), intrinsic defects in aged B cells substantially impair the influenza-specific response. Firstly, the decreased ability in CSR and somatic hypermutation of aged B cells results in lower antibody titers against pathogens (168, 169). The generated antibodies in the elderly are also less protective due to their lower affinity and neutralization capacity (170). Furthermore, a recent study investigating the effectiveness of influenza vaccination at different ages observed an age-dependent increase or decrease in DNA methylation at specific CpG sites (171). These age-related epigenetic alterations could often be linked to the low responsiveness of the subject to influenza vaccination, indicating that epigenetic remodeling upon aging negatively impacts the humoral response (172). In addition, the percentage of ABCs in aged individuals negatively correlates with the responsiveness to the influenza vaccine (173). Frasca et al. reported that ABCs secrete pro-inflammatory cytokines, but do not produce antibodies against influenza antigens in aged individuals (174). However, several studies showed that influenza-specific ABCs differentiate into protective antibody-producing cells in both mice and humans (141, 175). Although the exact role of ABCs in infectious diseases is still under debate, elevated percentages of CD21^-^ CD11c^+^ ABCs were also detected in severe COVID-19 cases compared to mild cases (176) and high levels of pro-inflammatory cytokines were negatively associated with the risk for pneumonia (177).

### B Cell Aging in Autoimmune Diseases

As described above in the section regarding ABCs, aging of the B cell repertoire contributes to autoimmunity (**Figure 1**). Autoreactive B cells are concluded to be responsible for the production of autoantibodies, the presentation of autoantigens and the secretion of pro-inflammatory cytokines (178). Upon aging, the level of self-reactive antibodies is elevated, resulting in the onset and development of autoimmune diseases (179). Since ABCs were observed to secrete autoantibodies, recent studies examined the association of this B cell subset with autoimmunity. In several autoimmune diseases, such as common variable immunodeficiency (CVID) (180), rheumatoid arthritis (RA) (181) and systemic lupus erythematosus (SLE) (148), the number of ABCs was significantly higher in both patients and autoimmune prone mice compared to healthy controls. Remarkably, Rubtsov et al. observed that the frequency of the ABC subset was significantly higher in aged female RA patients compared to young female patients and male patients of any age (28), suggesting that the previously described sex differences in ABCs contribute to the higher prevalence of autoimmune diseases in women relative to men (182). Due to the young age of investigated male and female SLE patients, peripheral ABCs in SLE were not detected by Rubtsov et al. Nevertheless, recent studies discovered the presence of ABCs, induced by TLR7 signaling, in lupus mice and patients (29, 183). Duffy et al. already reviewed that upregulated TLR7 signaling facilitated autoimmune responses (184), however, the exact mechanisms leading to TLR activation in autoimmune diseases remain unclear. Santiago-Raber et al. speculated that autoantibodies promote the uptake of self-RNA by DCs, resulting in the stimulation of TLR7 (185). Moreover, antibodies against self-DNA can give rise to the formation of immune complexes, which can subsequently activate TLR9, leading to pro-inflammatory immune responses (186). However, Nickerson et al. demonstrated that TLR9 can inhibit TLR7 and ABC development (187), indicating a regulatory role for the receptor. Nonetheless, the tolerance mechanism induced by TLR9 fails in ABCs due to the pro-inflammatory signals CD40L, IFN-γ and IL-21, resulting in autoinflammatory B cells (188).

### B Cell Aging in Atherosclerosis

As B cells play an important role in the lipid-driven, chronic inflammatory disease atherosclerosis (189), B cell aging is expected to also impact atherosclerosis development. Atherosclerosis, the main underlying cause of fatal cardiovascular events, leads to the formation of plaque in medium- to large-sized arteries (190). Rupture or erosion of an atherosclerotic plaque causes myocardial infarction or stroke. A hallmark step for the initiation of atherosclerosis is the retention and subsequent oxidation of circulating low-density lipoproteins (LDLs) in the subendothelial space of the arterial vessel wall. Oxidation of LDL (oxLDL) results in the formation of oxidation-specific epitopes (OSEs) (191, 192), which are recognized by the immune system and thus trigger a series of inflammatory responses starting with the recruitment of monocytes (193). In the intima, these monocytes differentiate into macrophages that phagocytose oxLDL. As a result of oxLDL uptake, macrophages convert into foam cells, which further attract immune cells to the lesion. Although these immune cells mainly include monocytes and T cells, B cells are also recruited to the vessel wall of the atherosclerotic plaque (194). B cells affect plaque formation by regulating T cell responses and producing antibodies, such as antibodies against OSEs (189). Palinski et al. reported that these anti-OSE antibodies are able to block the uptake of oxidized LDL, thereby suggesting an atheroprotective role for B cells (195). In addition, Caligiuri et al. observed a decrease in atherosclerotic lesion size upon transfer of splenic B cells to apolipoprotein E (ApoE) deficient mice (196). Although these results indicate that B cells are anti-atherogenic, several studies showed reduced atherosclerosis after B cell depletion (197, 198). These contradictory results can be attributed to differential effects exerted by different B cell subsets during atherosclerosis progression (199, 200).

B-1 cells confer an atheroprotective function by their anti-oxLDL IgM production (201). In line with these findings, Gruber et al. reported decreased atherosclerotic lesion size as a result of B cell-specific depletion of sialic acid-binding immunoglobulin-like lectin G (SIGLEC-G), which resulted in increased numbers of B-1 cells and elevated OSE-specific IgM plasma levels (202). Although B-1a and B-1b cells show similar anti-atherogenic effects (203, 204), B-1a cells can give rise to pro-atherogenic IRA B cells (61). IRA B cells secrete GM-CSF, which promotes T_H_1 skewing, thereby aggravating atherosclerotic lesion formation (205). Moreover, IRA B cells induce the expansion of infiltrating monocytes and neutrophils and thus promote inflammation (62, 206). In contrast to B-1 cells, Kyaw et al. observed increased atherosclerotic lesions after an adoptive transfer of 5 × 10^6^ B-2 B cells to B lymphocyte-deficient ApoE^-/-^ mice (207). Moreover, B-2 depletion as a result of BAFFR deficiency in ApoE^-/-^ mice reduced atherosclerosis progression (208, 209). Similar to these findings, inhibition of BAFFR with monoclonal antibodies, which similar to BAFFR deficiency leads to increased soluble BAFF levels, decreased atherosclerotic plaque formation (210). Interestingly, it has been shown that an antibody-mediated neutralization of BAFF in atherogenic diet-fed ApoE^-/-^ and LDLr^-/-^ mice promoted atherosclerosis progression (211). Similar to BAFFR inhibition, pro-atherogenic B-2 cell numbers were also decreased upon BAFF neutralization, thereby suggesting an anti-inflammatory role of BAFF independent of BAFFR signaling and B cells in atherosclerosis. As mentioned previously, B-2 cells are divided in FO B cells and MZ B cells. Tay et al., reported that mature B cell-specific Blimp-1 deficiency impaired plasma cell differentiation from FO B cells, resulting in decreased IgG levels and reduced atherosclerosis development in LDLr^-/-^ mice (212). In line with these findings, Douna et al. discovered that specific FO B cell inhibition by BTLA stimulation resulted in significantly reduced atherosclerosis (213). In contrast to FO B cells, MZ B cells exhibit atheroprotective properties by suppressing the proatherogenic T_FH_ response, thereby inhibiting atherosclerosis development (214). Nevertheless, the exact functions of the distinct B-2 subsets in atherosclerosis need to be further investigated. Apart from B-1 and B-2 cells, B_REGS_ have been identified in atherosclerosis (215). B_REGS_ exhibit anti-atherogenic functions *via* various mechanisms, including secretion of IL-10 and IL-35, inhibition of GC B cells, apoptosis induction of effector T cells, and stimulation of the atheroprotective molecule adenosine (82, 216–218).

**Figure 2:**
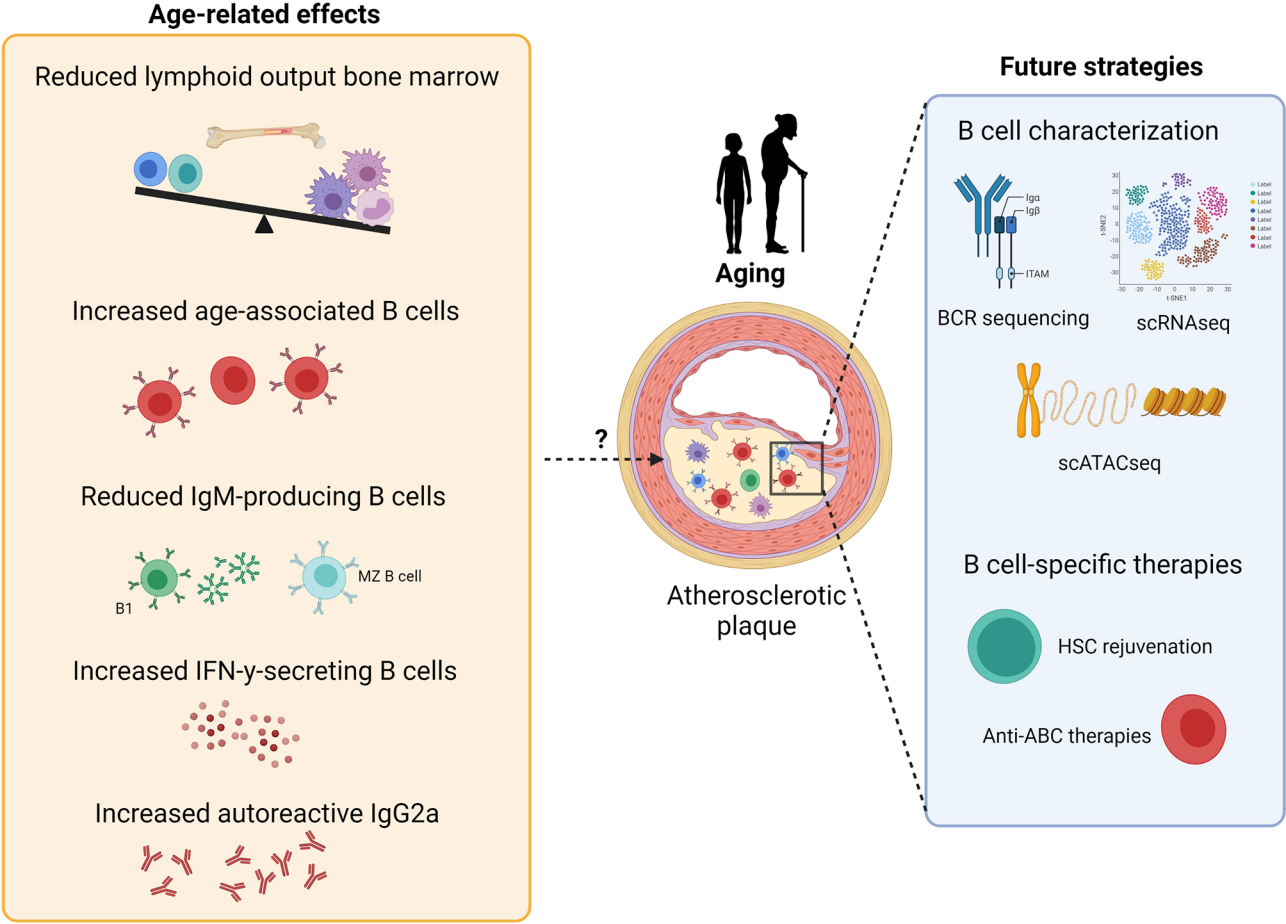
Potential effects of aging on atherosclerosis development and future strategies for B cell-specific treatment against atherosclerosis. B cell aging leads to reduced lymphoid output, reduced numbers of IgM-producing B cells and an increase in age-related B cell subsets, thereby resulting in both pro- and anti-atherogenic effects. Further characterization of B cells in atherosclerosis *via* single-cell RNA sequencing, BCR sequencing and single-cell assay for transposase-accessible chromatin sequencing should give further insights for the development of B cell-specific therapies against atherosclerosis, such as HSC rejuvenation and anti-ABC treatments.

Although studies investigating the effects of inflammaging on cardiovascular disease (CVD) are scarce, the increased prevalence of atherosclerosis upon aging suggests an important role for age-related immune alterations in atherosclerosis. Importantly, acute cardiovascular events mostly occur in the elderly and thus treatment of CVD patients occurs in the context of an aged immune system. Recent single-cell RNA sequencing (scRNAseq) studies have shown that B cells are present in atherosclerotic plaques of aged CVD patients (219–221) and an increased understanding of the contributions of age-induced B cell impairment to atherosclerosis progression is therefore crucial for atherosclerosis immunotherapy. Similar to the human atherosclerotic plaque, transcriptomic and single-cell analysis revealed that B cells are present in high-fat diet-induced lesions of young ApoE^-/-^ and LDLr^-/-^ mice (199, 222, 223). Although these studies show that the number of lesional B cells is limited, B cells have also been located in the perivascular adipose tissue surrounding the atherosclerotic aorta (224). In addition, several studies identified the presence of artery tertiary lymphoid organs (ATLOs) in close proximity of the aortic lesions of aged (75-85 weeks) ApoE^-/-^ mice (225, 226). ATLOs are lymphoid aggregates that form in the adventitia which contain high numbers of B cells, including B-1, GC and switched memory B cells (227). However, these ATLOs have only been identified in ApoE^-/-^ mice and single cell RNA sequencing analysis has not been performed on the atherosclerotic lesions of aged ApoE^-/-^ or LDLr^-/-^ mice to profile local B cells. During atherosclerosis progression, apoptotic and necrotic cells accumulate (228, 229) and enhance TLR7 and TLR9 ligands in the microenvironment. Possibly, this could promote the development of age-associated B cell subsets that contribute to atherosclerosis by the production of autoreactive IgG2a antibodies and the secretion of cytokines, such as IFN-γ and IL-17. As aging studies in atherosclerosis are limited, future research focusing on age-induced changes in B cell numbers, subsets and function in atherosclerosis, as indicated in **Figure 2**, will provide essential fundamental knowledge regarding disease etiology and may lead to novel targets to halt atherosclerosis progression.

## Future Strategies to Target the Aged B Cell in Chronic Inflammatory Disease

Due to the demographic shift towards an older population and the age-related increase in chronic inflammatory diseases, it has become a major public health priority to combat age-induced maladaptive immunity. Since aging reduces the lymphoid output of HSCs, HSC rejuvenation therapies, such as FOXOs and Cdc42 activity specific inhibitor (CASIN) (230), might be interesting therapeutic strategies to restore the balance between the myeloid and lymphoid pool. FOXOs are transcription factors which are involved in longevity and aging by regulating cell survival and growth (231). Specific deletion of FOXO1 and FOXO3a in HSCs induced apoptosis (232, 233), suggesting that FOXO overexpression might increase HSC survival. In addition to FOXOs, high Cdc42 activity was associated with HSC aging and treatment of aged HSCs with CASIN increased the common lymphoid progenitor pool, restored B cell numbers and elongated lifespan after transplant (234, 235). A more B cell-specific targeting drug, rituximab, proved to be effective in reducing RA and MS (236–238). Interestingly, Novikova et al. showed that rituximab also reduced atherosclerosis development in RA patients (239), indicating that B cell lymphopoiesis should be inhibited rather than stimulated in order to halt atherosclerosis progression in this setting. Notably, rituximab inhibits all CD20-positive B cells, including anti-inflammatory B-1 cells. Therapeutic strategies specifically targeting pro-inflammatory B cell subsets, such as ABCs and AABs, might therefore be more attractive to combat age-related immune diseases. NLRP3 inflammasome inhibition might be a potential candidate for this approach. Activation of the NLRP3 inflammasome is an important signaling pathway for ABC and AAB activation (126). Deficiency of this pathway in aged mice significantly reduced ABC and AAB numbers compared to wildtype aged mice (126). Notably, NLRP3 expression is not exclusively expressed by ABCs and AABs, underlining the urgency of the characterization of aged B cells in inflammatory diseases to develop specific anti-ABC therapies. Future studies identifying unique (age-associated) B cell subset markers, such as transcription factors, growth and survival receptors and immune checkpoints, are thus crucial to develop strategies to target ABCs or other pro-inflammatory B cell subsets in age-related diseases. Deep sequencing of the BCR could also provide important details for such cell-specific therapies (240). Recent BCR sequencing in young (20-45 years old) and old (60-80 years old) adults showed decreased BCR diversity, increased BCR clonality and different expression of BCR VHJ genes in aged patients (241), indicating that the aged immune cell repertoire might respond differently to pathogens and therapeutic agents. In addition, investigating autoantibodies produced by aged B cells might result in the identification of novel auto-antigens in chronic inflammatory diseases (242). Moreover, epigenetic alterations affecting B cell development, function and responses are observed with aging (243–245) and might contribute to age-associated changes in the B cell repertoire.

## Conclusion

With a rapidly rising life expectancy and demographic shift towards elderly, it is essential to enhance our understanding of age-associated immunity that causes disease susceptibility and mortality. In this review, we focused on age-associated alterations in B cell homeostasis in health and disease. Collectively, aging negatively affects the production of B cells in the bone marrow, resulting in decreased numbers of B-1 and B-2 cells. Moreover, antibody affinity and diversity are reduced upon aging, resulting in impaired antibody responses. Furthermore, aging induces the expansion of age-associated and adipose-resident age-related B cells, which contribute to inflammaging by the activation of pro-inflammatory T cell subsets and cytokine release. Although B cells are key drivers of autoimmune diseases, such as atherosclerosis, data on B cell aging in chronic inflammatory diseases is limited. Future studies identifying the aged B cell repertoire, including age-associated alterations in B cell numbers, subsets and antibody responses, are urgently needed in order to develop innovative B cell-specific therapies to combat chronic inflammatory diseases.

## Author Contributions

JM and ACF drafted the review and JM designed the Figures. JK, DT, and ACF provided critical feedback on the manuscript. All authors contributed to the article and approved the submitted version.

## Funding

This work is supported by the European Research Area Network (ERA-CVD B-eatATHERO consortium); Dutch Heart Foundation grant number 2019T107 to ACF and Austrian Science Fund (FWF) grant number I4647 to DT.

## Conflict of Interest

The authors declare that the research was conducted in the absence of any commercial or financial relationships that could be construed as a potential conflict of interest.

## Publisher’s Note

All claims expressed in this article are solely those of the authors and do not necessarily represent those of their affiliated organizations, or those of the publisher, the editors and the reviewers. Any product that may be evaluated in this article, or claim that may be made by its manufacturer, is not guaranteed or endorsed by the publisher.

## Abbreviations

APC, antigen presenting cell; ApoE, apolipoprotein E; ATLO, artery tertiary lymphoid organ; BAFF, B cell activating factor; BAFFR, B cell activating factor receptor; BCR, B cell receptor; B_REG_, regulatory B cell; BM, bone marrow; CASIN, Cdc42 activity specific inhibitor; CSR, class-switch recombination; CVD, cardiovascular disease; CVID, common variable immunodeficiency; DC, dendritic cell; FO, follicular; GC, germinal center; GM-CSF, granulocyte macrophage-colony stimulating factor; HSC, hematopoietic stem cell; IRA, innate response activator; MHC, major histocompatibility complex; MZ, marginal zone; oxLDL, oxidized low-density lipoprotein; pre-B cells, precursor B cells; pro-B cells, progenitor B cells; RA, rheumatoid arthritis; scATAC seq, single-cell assay for transposase-accessible chromatin sequencing; scRNAseq, single-cell RNA sequencing; SIGLEC-G, sialic acid-binding immunoglobulin-like lectin G; SLE, systemic lupus erythematosus; TD, T cell-dependent; T_FH_, follicular helper T cell; T_H_, T helper; TI, T cell-independent; TLR, toll-like receptor; T_REG_, regulatory T cell; VAT, visceral adipose tissue; APC, antigen presenting cell; BM, bone marrow; TH, T helper.
